# Family members’ lived experiences of non-compliance to psychiatric medication given to female adults living with depression

**DOI:** 10.4102/curationis.v44i1.2105

**Published:** 2021-01-07

**Authors:** Jeanne M. du Plessis, Marie Poggenpoel, Chris Myburgh, Annie Temane

**Affiliations:** 1 Department of Nursing, Faculty of Health Sciences, University of Johannesburg, Johannesburg, South Africa; 2 Department of Educational Psychology, Faculty of Health Sciences, University of Johannesburg, Johannesburg, South Africa

**Keywords:** adult females, depression, non-compliant, psychiatric medication, family members lived experiences

## Abstract

**Background:**

Family members face the burden of adult females living with depression who do not comply with psychiatric medication. Discomfort, tension, anxiety, frustration, and related feelings of hopelessness and dysfunction were identified by family members. There have also been records of financial problems, physical ill-health, limitations on social and recreational opportunities and a general deterioration in their quality of life. There is a shortage of published literature and information on the reasons for non-compliance to psychiatric medications. The existing body of information needs to be strengthened and future approaches encouraged. The study aimed to improve compliance of adult females dealing with depression to psychiatric medical treatment and the effect it has on family members caring for adult females living with depression.

**Objectives:**

To describe family members’ lived experiences of non-compliance to psychiatric medication by adult females living with depression.

**Method:**

A qualitative, exploratory, descriptive and contextual study design was used. A purposive sample of family members aged between 20 and 45 years was made. Data were collected by conducting eight in-depth, phenomenological interviews, and field notes were taken. The interviews focused on the central question: ‘Tell me your experiences of living with your wife, mother, sister and daughter living with depression and not taking their medication as ordered by the doctor?’ Tesch’s method for data analysis was used, and an independent coder analysed the data and met with the researcher for a consensus discussion of the results. Measures to ensure trustworthiness were applied and ethical principles were adhered to.

**Results:**

The three themes identified were: experienced psycho-social effects, experienced treatment refusal and experienced challenges in caring for adult females living with depression who are non-compliant to psychiatric medication. As a result, the absence of social help, disturbance of family working, shame, separation and troublesome conduct of the adult females who are non-compliant to psychiatric medication developed as principal subjects.

**Conclusions:**

The results demonstrated that family members experienced debilitation because they needed information about their relatives living with depression who are non-compliant to psychiatric medication. More information about the management of non-compliance of psychiatric medication was needed; a comprehensive awareness of the ramifications of the findings, treatment and care are required from mental health care professionals and service providers.

## Introduction

Compliance is the act of the patient complying with a prescribed medication plan (Cutler, Llimos & Frommer 2018:1–9). Medication adherence behaviour, therefore, refers to compliance by patients in taking their prescribed medications as instructed by their doctors and continuing to carry out their instruction throughout psychiatric drug therapy (Neus, Mangues & Tuneu 2016:1740–1754). Recent studies have found that compliance is a concept that describes the patient’s acceptance and understanding of the need to be treated willingly; it manifests as the degree of this acceptance and understanding with a positive or negative attitude towards drugs prescribed for the patient (Chukwujekwu & Adesokun 2017:1–10). Medication compliance is a worldwide concern to clinicians, the health care system, family members, caregivers and other stakeholders because of the mountain of evidence that non-compliance is general and associated with adverse outcomes and higher costs of care (Semahegn et al. 2018:1–5). Research (Elliot et al. 2016:747–758) has shown that compliance to medications for most chronic mental health conditions significantly drops after 6 months of treatment. Therefore, in psychiatry non-compliance is a significant problem (Rao et al. 2017:69–76). Sabin (2016:572–578) declared that between one-third and half of the medicines that are prescribed for long-term conditions are not used as recommended. In the case of depression, almost 76% of the patients become non-compliant to the medication within the first months of treatment (Ho, Jacob & Tangiisuran 2017:1–19).

### Problem statement

Non-compliance to prescribed medications is a remarkably common human experience (Malla, Joober & Garcia 2015:147–150). This behaviour and its impact on the family system, as well as disease management are magnified in chronic illnesses in psychiatric patients (Addo, Sencherey & Babayara 2018:1–10). For people with mental illnesses, non-compliance to psychiatric medication extensively adds to the burden of disease and leads to poorer long-term outcomes in these conditions (Alpak et al. 2015:151–156). Firstly, it is associated with a decreased likelihood of achieving a reduction in symptoms and recovery. Secondly, non-compliance increases the risk of relapse, hospitalisation and suicide attempts. Finally, the direct or indirect financial costs incurred by patients’ non-compliance to psychiatric medication are significantly higher because of increased treatment costs (Jawad et al. 2018:349–358).

Non-compliance to psychiatric medication may contribute to multi-faceted problems, so it becomes critically important to determine what effect it has on the family members and their experiences. Given the discussed problem statement, the researcher asked the following question:

What are family members’ experiences of non-compliance to psychiatric medication by adult females living with depression?

## Research purpose

The purpose of this research was to describe family members’ experiences resulting from non-compliance to psychiatric medication by adult females living with depression.

### Definition of key concepts

#### Experience

In phenomenological research, the researcher identifies the essence of human experiences concerning a phenomenon (Scharff 2019:1–11). The researcher brackets his experiences in order to understand those of the participants in the study (Clark & Creswell 2014:364–365). In this study, experience refers to the family members’ first-hand experience as a result of non-compliance to psychiatric medication by adult females living with depression.

#### Family members

Family members are persons who belong to a particular family, namely, a close relative (Kaakinen et al. 2014:4–6). In this study, family members are persons who care for each other in a family, which forms a basic social unit that is dwelling together (Brown 2010:1059–1077).

#### Non-compliance

Non-compliance is the act or state of not complying with or disregarding a prescribed medication plan (Cutler, Llimos & Frommer 2018:1–9). Moreover, the user does not adhere to prescribed treatment and does not return for any follow-ups. In this study, the family members’ experiences of non-compliance to psychiatric medication by adult females living with depression were explored and described.

#### Psychiatric medication

The term ‘psychiatric medication’, also known as psychotropic medication, is defined as medication that works by altering, blocking or enhancing levels of the brain’s naturally occurring chemical messengers, known as neurotransmitters (Bulow et al. 2016:820–828). A psychiatrist prescribes medication when symptoms of mental health or emotional illness are severe, persistent and interfere with normal functioning.

#### Depression

Depression is a common mental disorder that manifests with depressed mood, loss of interest or pleasure, decreased energy, feelings of guilt or low self-worth, disturbed sleep or appetite and poor concentration. At its worst, depression can lead to suicide (Malhi & Mann 2018:2299–2312). In this study, the focus was on family members’ experiences of non-compliance to psychiatric medication by adult females living with depression.

## Research design and method

A qualitative, exploratory, descriptive and contextual research design was utilised in this research (Burns & Grove 2013:23), embracing a phenomenological approach. Phenomenological studies look at human encounters through the portrayals given by the general population included (Creswell 2014:56).

### Population and sampling

The target population consisted of family members who have adult females who are non-compliant to psychiatric medication living with depression. Purposive sampling was utilised (Houser 2012:424). The criteria for consideration were family members with an adult female who is non-compliant to psychiatric medication living with depression, over 18 years old and who could communicate in English and/or Afrikaans. The unit manager of the mental health institution identified potential participants’ family members of adult females living with depression who are non-compliant to psychiatric medication admitted to the specific institution. The unit manager contacted the family members with adult females living with depression, non-compliant with psychiatric medication and invited them to take part in the study. After the family members agreed to participate in the study, their contact information was provided to the researcher on a confidential basis. The researcher met the families to arrange interviews with them. The setting for this study was in the institution itself in the ward where the adult female living with depression is admitted. The family members were requested to sign a consent form for the interview, as well as for the audio recording of the interview.

### Data collection

In-depth phenomenological interviews were conducted with one family member from each of the eight family members who cares for an adult female who is non-compliant to psychiatric medication and living with depression.

Open-ended questions were asked to all the family members. The central question asked to the family members was the following: ‘Tell me your experiences of living with your wife, mother, sister and daughter living with depression and not taking their medication as ordered by the doctor?’

Field notes were used as a data collection method and were taken during the interviews. These field notes consisted of personal, methodological, observational and theoretical field notes (Gray, Grove & Sutherland 2017:256–257; LoBiondo-Wood & Haber 2014:276; Polit & Beck 2017:22). Data saturation was reached when no new themes or categories emerged from the in-depth phenomenological interviews (Gray et al. 2017:254).

### Data analysis

The transcribed audio-recorded interviews and field notes were interpreted and investigated utilising Tesch’s descriptive analysis technique of open coding (Creswell 2013:155–156). The results of the analysis were discussed with an independent coder who had a PhD in psychiatric nursing and was experienced in qualitative research. Then, a consensus was reached on the themes of the results. The results were supported by adequate literature.

### Trustworthiness

Guba’s model of trustworthiness was applied in this study, and the four criteria of trustworthiness were used. These were credibility, transferability, dependability and confirmability (De Vos et al. 2011:419–420). Credibility refers to confidence of the truth in the data and interpretation of the data. The researcher invested time with the family members of the adult females who are non-compliant to psychiatric medication and living with depression, which offered an open door for a broad interaction as a natural process. Triangulation of information occurred through interviews, observation and field notes. Member checking was accomplished, as the members were given the consequences of the study to approve the right understanding of the interview, whilst an expert with a PhD in psychiatric nursing acted as an independent coder.

Transferability alludes to the degree to which the findings of the request have appropriateness in different settings, or with different members (Lincoln & Guba 1985:290). The demographics of the family members were extensively described. A thick description of the results of the interviews with verbatim quotations from the participants was given. Dependability is the measure to yield similar outcomes with the utilisation of the same research methodology in similar settings. An extensive description of the research methodology was given.

Confirmability is the objectivity or neutrality of data and interpretations (De Vos et al. 2011:419–420). A chain of evidence was provided throughout the entire research process. The interview transcripts, audiotapes and field notes were kept for this reason. An independent coder carried out a confirmability audit, and the researcher and independent coder were able to reach consensus regarding the confirmability of the data.

### Ethical consideration

Ethical approval to conduct the study was obtained from the Faculty of Health Sciences Research Ethics Committee, University of Johannesburg. The researcher adhered to the four ethical principles as indicated by Dhai and McQuoid-Mason (2011:13–14): autonomy, non-maleficence, beneficence and justice. Autonomy expounds that the researcher demonstrated regard for the participants and family members made their own choices to participate. Consent was obtained to do the research. Confidentiality was guaranteed, as information, that identified the participants were not revealed. The aggregate of potential benefits to a participant and the knowledge accrued ought to exceed the danger of damage (Kvale & Brinkmann 2014:68–72). Participants were not put in danger in this research study.

Non-maleficence implies that an individual must not cause hurt: for example, interviewing participants and withholding information without giving an assurance of security. Debriefing sessions were accessible whenever required. Beneficence suggests representing the benefit of the participant (Gray et al. 2017:163–168). The researcher gave feedback that there were no financial benefits or damage foreseen for the participants. Recounting their experiences benefitted other individuals too, who were in a similar circumstance, and added to the body of scientific information. The researchers dealt with the interviews scrupulously to avoid any uneasiness and were conscious of indications of distress (Gray et al. 2017:163–168). Justice identifies with decency in the determination of individuals included, and dispersion of the weights and benefits of the research. Participants were selected according to the relevant criteria of the study.

## Findings

A description of the demographics of the participants and themes of family members’ experiences of non-compliance to psychiatric medication by adult females living with depression (Du Plessis 2019:35–36) follows.

### Description of the demographics of the participants

The participants who were interviewed consisted of five daughters, with ages ranging from 26 to 45 years old, two husbands who were between 38 and 40 years old, and a sister who was 45 years old. The age range thus varied between 26 and 45 years. Table 1 presents the demographic data of the eight in-depth phenomenological interviews with family members as participants (Du Plessis 2019:35–36).

**TABLE 1:** Demographic data of participating family members.

Participants number	Gender	Age	Level of education	Relationship to adult females living with depression who are non-compliant to psychiatric medication	Years caring for adult female living with depression who are non-compliant to psychiatric medication
Participant 1	Female	26	Grade 12	Daughter	8 years
Participant 2	Female	41	Grade 12	Daughter	5 years
Participant 3	Male	38	Grade 12	Husband	10 years
Participant 4	Female	45	Grade 12	Daughter	9 years
Participant 5	Female	40	Grade 12	Daughter	10 years
Participant 6	Female	43	Grade 12	Sister	2 years
Participant 7	Female	35	Grade 12	Daughter	4 years
Participant 8	Male	40	Grade 12	Husband	10 years

*Source*: Du Plessis, J.M., 2019, Family members’ experiences of adult females living with depression non-compliant to psychiatric medication. M Cur Psychiatric Nursing Thesis, University of Johannesburg, Johannesburg, p. 35

### Family members’ experiences of adult females living with depression non-compliant to psychiatric medication

Findings of the interviews: Themes from experiences of family members’ living with adult females with depression and are non-compliant to psychiatric medication.

Three themes were identified from the data (Du Plessis 2019:37–39). Table 2 presents the findings of the interviews.

**TABLE 2:** Experiences of family members’ living with adult females with depression and are non-compliant to psychiatric medication.

Theme	Description
Theme 1	Family members experienced psycho-social effects of adult females living with depression who were non-compliant to psychiatric medication
Theme 2	Family members experienced treatment refusal by adult females living with depression who were non-compliant to psychiatric medication
Theme 3	Family members experienced challenges caring for adult females living with depression who were non-compliant to psychiatric medication

*Source*: Adapted from Du Plessis, J.M., 2019, Family members’ experiences of adult females living with depression non-compliant to psychiatric medication, M Cur Psychiatric Nursing Thesis, University of Johannesburg, Johannesburg, pp. 37–39

#### Theme 1: Family members’ experienced psycho-social effects of adult females living with depression who are non-compliant to psychiatric medication

Whilst staying with adult females living with depression who are non-compliant to psychiatric medication, most family members reported that they experienced disruptive behaviour from these women as they drank alcohol and used drugs. Family members also reported that they did not get any support from their extended family members and relatives. Participants reported that adult females living with depression isolated themselves at home.

Family members experienced uncontrolled and disruptive behaviour from adult females living with depression who are non-compliant to psychiatric medication.

In interviews conducted in this study, family members of adult females living with depression mentioned that it was difficult for them to stay with these women when they were at home under the family members’ care as they behaved badly and uncontrollably. Participants shared the following experiences:

‘If she does not take her medication she goes off the chart go to different places and sometimes has a car and still drive so she would get in the car and end up somewhere. Sometimes and uhhhm the problem is alcohol. We have a cabinet that we can lock now so she can get in near meds or alcohol stuff like that. She will grab it she will hide it from us when I’m not there stuff like that so you need to be more observant.’ (P1, daughter, 26 years old)

‘Firstly she was not taking her medication and swearing at us doing bad things like taking off her clothes and talking to herself and not listening to us.’ (P4, daughter, 45 years old)

Family members reported that when the adult females living with depression, who are non-compliant to psychiatric medication, were at home, they used drugs, such as alcohol and dagga. Participants thus experienced problems because of the effects of substance abuse and alcohol. This was evidenced by the participants who said that:

‘Like we went out at night had a few drinks that you know the next day she has a hangover is basically that kind of effect.’ (P3, husband, 38 years old)

‘Her behaviour becomes out of control on top of that she likes to drink alcohol.’ (P8, husband, 40 years old)

Family members revealed that adult females living with depression became aggressive towards them for no apparent reason. Participants indicated that the adult females living with depression would become verbally aggressive when they do not take their medication. Participants said:

‘She talks to herself she will, she will almost like wreck the car and she will scream sometimes. How can I say she will be like a child is she can’t get what she wants she will throw tantrum stuff like that.’ (P1, daughter, 26 years old)

‘First of all she is very rude and verbally aggressive.’ (P7, daughter, 35 years old)

Family members reported that the adult females living with depression isolated themselves at home and seldom talked to anyone in the family. Family members reported that:

‘There is times where she isolates herself totally. But I have to go and check if things are okay you know. When she is quiet, I know something is up.’ (P7, daughter, 35 years old)

‘She mostly isolates herself and sleeps a lot I mean hours and hours on end.’ (P8, husband, 40 years old)

In this study, family members reported that they experienced lack of support from other family members and the healthcare system. This resulted in feelings of loneliness and abandonment. They shared:

‘It seems like I am running out of options. I feel that I maybe should just do more and I do not know what to do really I don’t know.’ (P2, daughter, 41 years old)

‘I mean my family, my family does not want to support me and also my friends the bad thing is I have no support system at all I have to deal with everything on my own.’ (P8, husband, 40 years old)

#### Theme 2: Family members experienced treatment refusal by adult females living with depression who are non-compliant to psychiatric medication

Complying with medication was the only way that individuals living with mental illness could maintain their mental stability. However, if the individuals living with mental illness are non-compliant with their prescribed medications, they might present with depressive symptoms. This was predominantly caused by avoidable factors, but because of a lack of knowledge and different belief systems, individuals living with mental illness who were determined to stop taking their medication affected the family dynamics. The following quotations demonstrate this:

‘How can I say it consumes you and then you also need to find something and pull through and take her with you so you need to take course and you need to have direction in life.’ (P1, daughter, 26 years old)

‘I have reached a point that I do not know what to do anymore. I have tried everything for her to be more compliant.’ (P7, daughter, 35 years old)

Family members reported that adult females living with depression deliberately refused to take prescribed medications, or stopped taking their medication as a result of unforeseen circumstance out of the family members’ control. The family members in this study also shared experiences that the adult females living with depression did not want to take their medications as they believed they were no longer mentally ill. Family members reported that the adult females refuse to be supervised in taking their medications. They stated that adult females living with depression defaulting on medication resulted in relapse, and these women then once again presented with depression. Participants said:

‘She refuses to take her treatment when at home; she slapped me while I was advising her to take her medication.’ (P6, sister, 43 years old)

‘I don’t think there’s much I can do. I have tried everything exhausted all avenues spoke to family members spoke to doctors. She just does not want to take medication when she’s at home and how can you convince a person that has fixed believed that pills are for crazy people.’ (P8, husband, 40 years old)

#### Theme 3: Family members experienced challenges caring for adult females living with depression who are non-compliant to psychiatric medication

Most family members reported that they experienced fear, distrust and felt insecure when they were staying with the adult females living with depression. They reported that this affected them psychologically. They also reported that they did not trust that the adult females living with depression could function on their own. The participants mentioned that they were struggling at times to cope with the adult females living with depression at home.

Family members experienced feelings of persecution related to suspicion, burden and fear. Participants reported that they were afraid of the behaviour of the adult females living with depression when they were at home and did not comply with their medication. They said:

‘Sometimes I feel like maybe I am not doing enough, just I don’t know I want to blame myself it’s just confusing.’ (P2, daughter, 41 years old)

‘I am alone and sometimes when things get out of hand with my mother I run to my room and locked the door.’ (P7, daughter, 35 years old)

According to participants, their relative’s depression was a major barrier for optimal adherence to medication. The following comments made by participants describe how their relative’s depression was a major barrier for optimal adherence to medication:

‘That’s what I do focus on the good. It wasn’t always like that most, most of the time when I was younger I focused on the bad stuff that’s why I went to therapy and that started to rewrite your brain.’ (P1, daughter, 26 years old)

‘Aggggg, I just feel tired sometimes it drains me.’ (P5, daughter, 40 years old)

Participants were of the opinion that they had difficulty in coping with stressful situations whilst staying with the adult female living with depression. The participants reported their experiences of a lack of coping mechanisms as follows:

‘I feel that I maybe should just do more I do not know what to do really I don’t know.’ (P2, daughter, 41 years old)

‘I really do not have any coping mechanisms. I am alone.’ (P7, daughter, 35 years old)

It is important for family members of adult females living with depression to have the necessary strength to take care of them. However, it was difficult for the family members to provide care for these women as they encountered problems such as tiredness and exhaustion because of adult females living with depression displaying uncontrollable behaviour.

Physical exhaustion as a result of the nature of continuous care was also experienced. Most family members experienced physical exhaustion because of a lack of rest from pursuing and restraining someone who is restless. Participants explained:

‘I don’t sleep at night, thinking about her, this is really difficult for me, I tried to talk to our father about her behaviour but he does not listen to me.’ (P6, sister, 42 years old)

‘Then I always have to rush home and defuse the situation. This is really causing me exhaustion physically and mentally. At night, I also lay awake and make sure my wife is OK and this causes me not to sleep.’ (P8, husband, 40 years old)

Stigma was another theme that emerged. Family members caring for adult females living with depression, who are non-compliant to psychiatric medication reported that they did not only bear the burden of caring for these women and incurred financial expenses, they were also ostracised and isolated. The family members expressed that there was still a lot of stigmas associated with mental illness and they attributed this to a lack of knowledge on the part of their relatives and the community at large. They expressed their experiences as follow:

‘I have noticed because the stigma of your grandmother is mad you know that the kids think even if you teach them they would say mama I understand but my friends will not understand if grannie is like this’. ‘I have kids and they become embarrassed.’ (P5, daughter, 40 years old)

‘I think they are still having a grudge because of her mental illness. They are kind of stigmatising her this makes me hopeless I did not raise my children like this.’ (P7, daughter, 35 years old)

Family members expressed the need for them to be properly trained to deal with psychological problems. Participants complained that they did not know what their relatives were suffering from because it had never been explained to them. They said that they just knew that their relatives had a psychiatric problem, but they were not aware of the specific diagnosis. Most of the participants said that they get so overwhelmed by the condition that they end up ignoring the patient or ill-treating them. Participants said:

‘You’re right I am very emotional when talking about it. My family pushed me one side because of my mother’s behaviour at times. Ja that is one of the contributing factors to my emotional state.’ (P7, daughter, 35 years old)

‘To be honest I do not have any support from family or friends because they are scared at times and they don’t know her condition. Also the health care system just gives you medication but not ways on how to deal with the situation when it occurs so I mostly rely on what the internet says.’ (P8, husband, 40 years old)

## Discussion of findings

The discussion of findings will be based on the three identified themes: family members experienced psycho-social effects, lack of support and challenges caring for adult females living with depression who are non-compliant to psychiatric medication.

### Family members experienced psycho-social effects of adult females living with depression who are non-compliant to psychiatric medication

Uncontrolled and disruptive behaviour was highlighted by Rogers (2013:405–412) stating that many families have the frightening experience of realising they have no control over adult females living with depression, especially if the woman is aggressive towards them. Behere, Basnet and Campbell (2017:457–463) verified that mental illness could result in devastation within the family. The destructive behaviour of the person with mental illness can cause trauma, tension, guilt and bitterness. It can tear marital relationships to shreds and cause resentment and even hatred between siblings.

The findings of this study had some similarities to the study of Bailey et al. (2017:140–146), who also found that most psychiatric patients who are under the care of their families suffer from financial instability as a result of using tobacco, alcohol and drugs, which makes them aggressive towards their family members. In such families, individual family members seem to become restricted in expressing their needs, feelings and wishes (Schultz & Alpaslan 2016:90–94). A change in the family structure becomes noticeable, characterised by distorted patterns of communication and a lack of understanding amongst family members (Brage et al. 2018:1–13).

In a study by Pompili et al. (2017:175–179), they found that 60% of psychiatric patients show aggressive behaviour towards their caregivers, and such behaviour was a prevalent problem amongst adult females who were non-compliant to their psychiatric medication. Recently, concerns about the impact of family aggression and violence have increased (McCann et al. 2017:11).

Social connections to others, especially family members and friends, are critical features of life that provide a variety of social support in the form of assistance with routine activities, comfort and companionship. These connections are especially helpful during crises, and they reduce isolation (Taylor, Taylor & Chatters 2016:443–461). Increasing evidence confirms the negative effects of isolation on a person’s physical and mental health and well-being (Berkman, Kwachi & Glymoche 2014). Despite a significant body of research on isolation and its effects, very little of this work specifically focuses on the experiences of family members of mentally ill persons (Lavoie 2018:601–626).

Shamsaei, Cheraghi and Basshirian (2015:1–7) added that carers experienced a lack of support in dealing with their relatives’ problems. The social support needs expressed by these carers were categorised into informational, emotional and professional support (Kaakinen et al. 2014:2540–2600). The quality of support varies and many family members feel marginalised by services that seem unaware of their significant contribution as informal caregivers (Bauer & Alfonso 2015:113–145).

Disarray and family complication was found in family member’s home condition in the light of this research by family members caring for adult females who are non-compliant to psychiatric medication. In this research, the overwhelming instability experienced by family members highlighted their difficulty in adequately dealing with their feelings. There is an emotional response to the conflict in relationships and difficulty in naming or fully experiencing emotions (Aguirre & Galen 2013:23).

Subjective imbalance shown by family members caring for adult females who are non-compliant to psychiatric medication living with depression prompted scenes of experienced separation. The research identified that family members were incredibly resentful to analysis and judgement, and felt unsupported by other relatives.

Social imbalance was seen when the family members felt overpowered by circumstances. The adult females who were non-compliant to psychiatric medication behaved rashly to express their feelings, with damaging consequences leading to alcohol and drug misuse.

### Family members experienced treatment refusal by adult females living with depression who are non-compliant to psychiatric medication

Akbari et al.’s (2018:329–337) study also found that adult females living with depression and refusing psychiatric medication led to developments that affected family caregiving. Firstly, the de-institutionalisation movement has been moving people with severe and persistent mental illness from hospitals to the community. The movement has accelerated given the growing concern for the non-therapeutic aspects of hospital care and the civil rights of the patients. Medications for depression have had a dramatic effect on some patients living with mental illness, by reducing behaviours that were previously difficult to manage.

According to Crowe, Wilson and Inder (2011:894–903), defaulting treatment can be in the form of not taking the medication at the recommended dose and frequency, not taking the medication at all, and irregular attendance to follow-up appointments or not attending at all. The most important and highly encountered form of defaulting medication is where a patient does not follow the recommended dose and frequency (Mert et al. 2015:87–93).

### Family members experienced challenges caring for adult females living with depression who are non-compliant to psychiatric medication

Some family members experienced feelings of persecution related to suspicion, burden and fear. Participants reported that they were afraid of the behaviour of the adult females living with depression when they were at home and did not comply with their medication. This is supported by Lee and Selart (2015:153–159), stating that just one betrayal may create distrust and once established, distrust is extremely resistant to change. Family members reported tension, stress, anxiety, resentment, depression with accompanying feelings of hopelessness and powerlessness, a sense of entrapment, a disruption in their family life and relationships, financial difficulties, physical ill-health, restrictions in social and leisure activities, and an overall decrease in the quality of life as a result of having a relative living with mental illness.

According to participants, their relative’s depression was a major barrier for optimal adherence to medication. According to Paiva et al. (2017:34–42), depression is associated with feelings of hopelessness and loss of the will to care for the self. Family members experienced hopelessness in caring for adult females living with depression who are non-compliant to psychiatric medication. Hopelessness increases the risk of emotional maladjustment for family members (Assari & Lankarani 2016:1–6). Caring for a family member with a chronic mental disorder can be quite daunting, and there is the tendency for family members to feel oppressed by the associated tasks involved (Aloba et al. 2016:18–25).

Lesesselo, Kajula and Malima (2016:1–13) noted that parents of psychiatric patients attempted to avoid conflict or confrontation with the affected person as a way of providing care and coping. These studies suggest that these families use cognitive as well as behavioural strategies to cope with the burden, stressors and the difficult realities of their loved one’s illness.

The impact of caregiving on the well-being of family members has been studied in different communities across the world (Van Deventer & Wright 2017:2–7). Swaroop et al. (2013:30) established that caregiving can be an emotionally and physically draining experience for family members. These caregivers often have high rates of depression when compared to the general population. Some family members expressed a sense of hopelessness, physical exhaustion and despair.

Mental illness is an exceedingly stigmatised condition within certain communities, making it challenging for individuals to seek effective treatment (Rossler 2016:1250–1253). The consequences of such stigma can have lifelong effects for the individuals, the families and the communities (Dieujuste 2016:201–206). The family members said that society still uses labels, such as saying someone is ‘mad’ and shunning mentally ill patients (Peggy 2011:6–28). This was also related to beliefs about the causes of mental illness, such as it being a punishment for wrongdoings by the ancestors (Quinn & Knifton 2014:554–559). Participants were of the opinion that mental illness exposed them for their wrongdoings.

According to Alberts and Simpson (2015:2753–2767), family members play a significant role in supporting a loved one during a mental health crisis. This claim is also supported by MacCourt and Family Caregivers Advisory Committee (2013:n.p.), stating that an estimated 2% – 3% of the adult population provides informal care and support to a family member living with a mental health issue. Lesesselo et al. (2016:2–10) found that family members experienced a lack of support in dealing with their relatives’ problems. The social support prerequisites voiced by these family members were classified into informational, emotional and professional support.

## Recommendations

Caring for adult females who are non-compliant to psychiatric medication living with depression causes pressure and conflict for both the family members and the adult females. More help for family members’ mental health is recommended. This help could be through engaging family members and the adult females who are non-compliant. The significant revelation from this research is that relatives experienced a lack of knowledge and the skills to deal with adult females living with depression, who are non-compliant to psychiatric medication. This prompted the difficult process of briefing and adequately equipping the family members to deal with their own emotional well-being more successfully, whilst dealing with females living with depression who are non-compliant to psychiatric medication. More studies of family members’ experiences of caring for adult females living with depression and non-compliant to psychiatric medication, is required to propose a comprehensive framework for the treatment and care of such patients.

## Limitations of the study

This study was conducted in a psychiatric hospital. Therefore, the findings may not be applicable to family members with adult females who are non-compliant to psychiatric medication living with depression in the community.

## Conclusion

This research study had the respectable objective of starting to gather information on the understandings and to talk about family members’ experiences of psychiatric medication non-compliance by adult females living with depression. The sample is small and may not be reflective, but there was a relatively consistent representation of the lack of comprehension of the condition, a significant family tension, and little corroboration of a close working relationship between family members of adult females living with depression who are non-compliant to psychiatric medication and mental health care providers. The situation of all family members interviewed, it indicates that it might also be helpful to observe the evidence from literary sources more closely and to include family members more actively in care of the adult females living with depression who are non-compliant to their psychiatric medication. If what many participants in this study say is recognised to be accurate, we have a way to go forwards to promote the best possible relationship with family members and the mental health care system, and to better equip these family members and adult females living with depression who are non-compliant to their psychiatric medication.
